# deFUME: Dynamic exploration of functional metagenomic sequencing data

**DOI:** 10.1186/s13104-015-1281-y

**Published:** 2015-07-31

**Authors:** Eric van der Helm, Henrik Marcus Geertz-Hansen, Hans Jasper Genee, Sailesh Malla, Morten Otto Alexander Sommer

**Affiliations:** Novo Nordisk Foundation Center for Biosustainability, Technical University of Denmark, 2970 Hørsholm, Denmark; Department of Systems Biology, Center for Biological Sequence Analysis, Technical University of Denmark, 2800 Lyngby, Denmark; Novozymes A/S, 2880 Bagsværd, Denmark; Department of Systems Biology, Technical University of Denmark, 2800 Lyngby, Denmark

**Keywords:** Functional metagenomics, Web services, Visualization, Sequence analysis, Gene ontology

## Abstract

**Background:**

Functional metagenomic selections represent a powerful technique that is widely applied for identification of novel genes from complex metagenomic sources. However, whereas hundreds to thousands of clones can be easily generated and sequenced over a few days of experiments, analyzing the data is time consuming and constitutes a major bottleneck for experimental researchers in the field.

**Findings:**

Here we present the deFUME web server, an easy-to-use web-based interface for processing, annotation and visualization of functional metagenomics sequencing data, tailored to meet the requirements of non-bioinformaticians. The web-server integrates multiple analysis steps into one single workflow: read assembly, open reading frame prediction, and annotation with BLAST, InterPro and GO classifiers. Analysis results are visualized in an online dynamic web-interface.

**Conclusion:**

The deFUME webserver provides a fast track from raw sequence to a comprehensive visual data overview that facilitates effortless inspection of gene function, clustering and distribution. The webserver is available at cbs.dtu.dk/services/deFUME/and the source code is distributed at github.com/EvdH0/deFUME.

**Electronic supplementary material:**

The online version of this article (doi:10.1186/s13104-015-1281-y) contains supplementary material, which is available to authorized users.

## Findings

### Background

Functional selection represents a powerful technique for discovery and functionally validated annotation of genes [1, 2]. The technique relies on the expression of randomly cloned genomic or metagenomic DNA, typically as short (1–3 kb) fragments of DNA into an expression vector. The expression library is subsequently transformed into a suitable host where the desired functionality can be selected. DNA inserts from clones exhibiting the desired phenotype can be sequenced, enabling functional isolation of novel genes. The approach has been applied for identification of genes from complex metagenomic sources and examples include DNA polymerases [3], antibiotics resistance genes [1, 2, 4, 5], xenobiotic degradative enzymes [6], and more [7].

Most commonly, inserts of 1–3 kb are sequenced either by next generation sequencing [4, 8] or by conventional bi-directional Sanger sequencing, followed by base-calling, quality trimming of reads and assembly of reads into contigs. Finally data analysis is performed including BLAST searches and other functional annotations. Whereas hundreds to thousands of clones can be easily generated over a few days of experiments, analyzing the data is time consuming and constitutes a major bottleneck for experimental researchers in the field. To address this challenge we developed deFUME; an easy-to-use web server that automatically processes and annotates large amounts of sequencing data obtained through functional selections.

## Implementation

As input, deFUME accepts nucleotide sequences generated from next generation sequencing technologies as well as raw reads from Sanger sequencing. Sequences can be uploaded via the submission page either as preassembled projects, when using next generation sequencing data in. FASTA format, or as raw Sanger sequencing chromatograms (.ab1 format). For Sanger sequencing chromatograms, base-calling and assembly of the resulting reads is carried out with Phred [9] (default parameters) and Phrap [10] (using the non-default parameters -minscore 25, -trim_score 20, and -min_match 20 to reflect medium stringency cutoffs in order to ensure good quality assemblies of Sanger reads) respectively. Prior to assembly the vector sequence is optionally masked in the reads using Cross_match (using -minmatch 12 -minscore 20) [10] (Additional file 1: Figure S1).

Open reading frames (ORFs) are predicted using MetaGeneMark [11] (default parameters) from the resulting assembly (generated by Phrap or user input). The translated ORFs are aligned to the nr protein database using BLASTp (reporting only the 25 most significant hits with a minimal E value of 0.001) [12] and submitted to InterPro [13] (default parameters). The InterPro database contains signatures of known proteins families that can be queried to functionally characterize new sequences. To ensure using the most recent databases, InterPro is accessed using the simple object access protocol (SOAP) via InterProScan 5 [14].

**Fig. 1:**
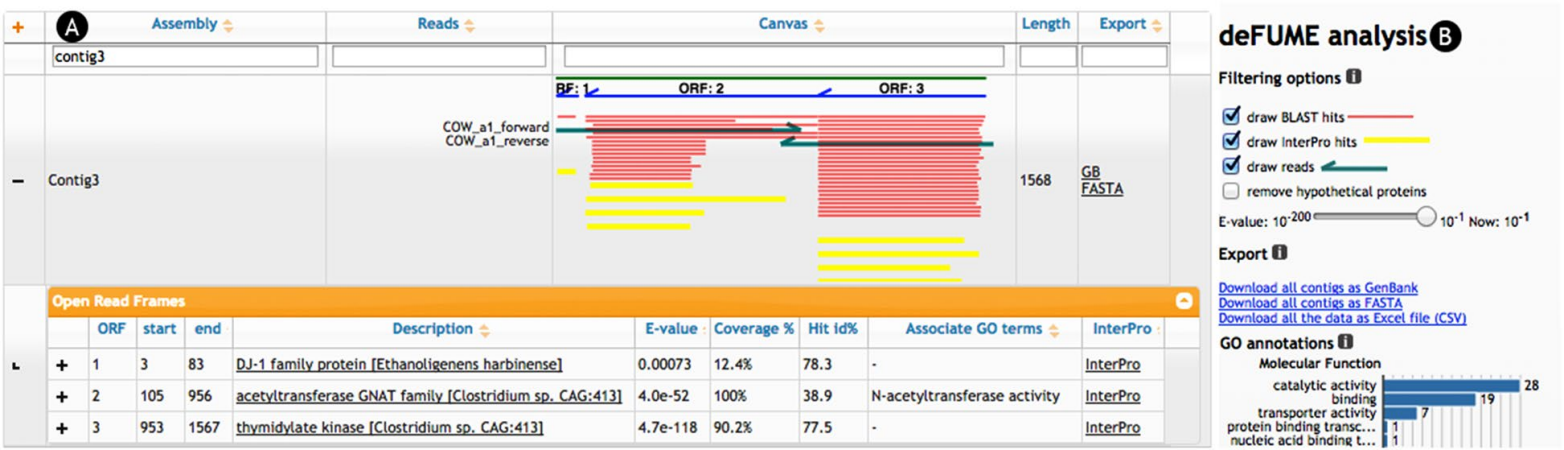
The deFUME output page. **a** Screenshot from the deFUME interactive output showing two Sanger reads (*dark green*) assembled into a 1,568 bp contig (*green*) by Phrap. deFUME annotated three open reading frames; ORF1 is annotated by BLASTp as a DJ-1 family protein, ORF2 as an acetyltransferase and ORF3 as a thymidylate kinase. The “Coverage %” *column* shows that only ORF2 is a complete gene, indicating the phenotypic activity from this clone likely arises from ORF2. All ORFs contain multiple Interpro hits (*yellow*) that can be inspected in detail by clicking on the “Interpro” link which launches the native Interpro webpage. **b** deFUME analysis toolbox. The data can be filtered and manipulated by turning on and off different levels of metadata, filter on E value and filter on specific GO terms.

The deFUME output page (Fig. 1a) is an interactive table showing the sequencing reads, the assembled contigs, predicted ORFs, BLASTp hits and InterPro functional data. The user can highlight hits and filter (Fig. 1b) the data by parameters such as BLAST E value, specific GO terms and removal of hypothetical protein homologs. Finally, the user can export selected contigs in FASTA, Genbank and CSV file formats.

The back-end pipeline is written in the programming language Perl (version 5.8.7) and PHP5 and the front-end visualization in a combination of JavaScript and HTML5 using the D3js, jqGrid and jQuery packages. The access to the webserver is free and unlimited for all academic users with a maximal data upload time of 2 min per job and a maximal job runtime of 24 h. The source code is freely available at https://github.com/EvdH0/deFUME for continuous improvement and development by the community.

## Results and discussion

To demonstrate and test the deFUME web server, we analyzed Sanger sequencing data derived from a functional metagenomics selection for genes conferring tolerance of *E. coli* to high levels of lysine. Briefly, metagenomic DNA derived from cow fecal matter was mechanically sheared and subsequently cloned and expressed in *E. coli.* The resulting cell library was subjected to high lysine levels on Luria–Bertani agar plates (Additional file 1). The inserts of 80 individual colonies tolerant to high lysine levels were sequenced. The resulting 160 raw Sanger sequencing chromatograms were submitted as.ab1 files to the deFUME web server. In less than 2 h, all reads were trimmed and assembled, resulting in 69 unique contigs, 117 predicted ORFs, 134 GO annotations and 622 InterPro functional predictions. A screenshot of the output page is shown in Fig. 1, displaying one of the 69 inserts. In this particular insert, deFUME predicted three ORFs with a sequence coverage (calculated by dividing the protein sequence length of the predicted ORF by the length of the best BLASTp hit) of 12, 100 and 90%. Together with an E value of 4^−52^ this indicates that ORF 2 likely encodes the mechanism for lysine tolerance. The translated ORF is annotated by BLASTp as an “acetyltransferase GNAT family” protein catalyzing the acetylation of the nitrogen group of lysine, thus providing tolerance to high levels of lysine. The example demonstrates the ability of deFUME to accelerate the overall process of going from experimental raw data to functional annotation.

## Conclusions

deFUME is the first web server to integrate all steps from sequencing read assembly to a comprehensive visual output for functional annotation of metagenomic insert libraries. Additionally, deFUME reduces the hands-on time required for analysis compared to packages like CLC Main Workbench (CLC Bio, Aarhus, Denmark) and Mobyle [15], where the user has to transfer the intermediate data from one tool to the other. Furthermore, the data integration and visualization of deFUME is substantially more advanced compared to the current state-of-art by providing interactive exploration of heterogeneous data.

## Availability and requirements

Project name: deFUME.

Project home page: http://www.cbs.dtu.dk/services/deFUME.

Operating system(s): Platform independent.

Programming language: Perl, PHP, Javascript, HTML5.

Other requirements: Browser supporting HTML5.

License: Creative Commons BY 2.0

Any restrictions to use by non-academics: no.

## Additional file

Additional file 1. Supplementary information.
