# Supplementary material for: deFUME: Dynamic exploration of functional metagenomic sequencing data
Source: BMC Res Notes. 2015 Jul 31;8:328. doi: 10.1186/s13104-015-1281-y (PMC4520277; doi:10.1186/s13104-015-1281-y)
Supplement: Additional file 1. — Supplementary information. [file 13104_2015_1281_MOESM1_ESM.pdf]

Supplementary information

deFUME: Dynamic Exploration of Functional Metagenomic Sequencing Data

Eric van der Helm[1,*], Henrik Marcus Geertz-Hansen[1,2,3], Hans Jasper Genee[1], Sailesh Malla[1], and Morten O. A. Sommer[1,4]

[1] Novo Nordisk Foundation Center for Biosustainability, Technical University of Denmark, DK-2870 Hørsholm, Denmark

[2] Center for Biological Sequence Analysis, Department of Systems Biology, Building 208, Technical University of Denmark, DK-2800 Lyngby, Denmark

[3] Novozymes A/S, Krogshøjvej 36, DK-2880 Bagsværd, Denmark

[4] Department of Systems Biology, Technical University of Denmark, DK-2800 Lyngby, Denmark

[*]To whom correspondence should be addressed.

Functional metagenomic selection for genes conferring lysine tolerance

A metagenomic expression library of metagenomic DNA extracted from a cow fecal sample was constructed as described previously [1]. Briefly the procedure involves i) isolation of total DNA from 5 g of fecal matter using the PowerMax Soil DNA Isolation Kit (Mobio Laboratories Inc.), ii) fragmentation of extracted DNA by sonication into pieces of an average size of 2 kb using a Covaris E210 (Massachusetts, USA) and iii) blunt-end cloning into an expression plasmid (Figure S2). In this study the cloning vector pZE21 [2] was used. On the basis of the determined library sizes and titers of the frozen library stocks, 10^6 cells (i.e., 100 µl of the library stock) were plated out on LB agar supplemented with kanamycin (50 µg/ml) and L-lysine at the selective concentration (14 g/L). Plates were incubated at 37°C for 2–3 days and growth of colonies (likely lysine-tolerant clones) was assayed after 48-65 h of incubation. 80 clones tolerant to L-lysine (14 g/L), were chosen and their metagenomic inserts were sequenced by Sanger using forward primer (pZE21_F: 5'-ATC AGT GAT AGA GAT ACT GAG CAC -3') and reverse primers (pZE21_R: 5'-TTT CGT TTT ATT TGA TGC CTC TAG -3') annealing to the pZE21 expression vector backbone. The resulting 160 raw sequencing chromatogram files were submitted to the deFUME web-server and analyzed.

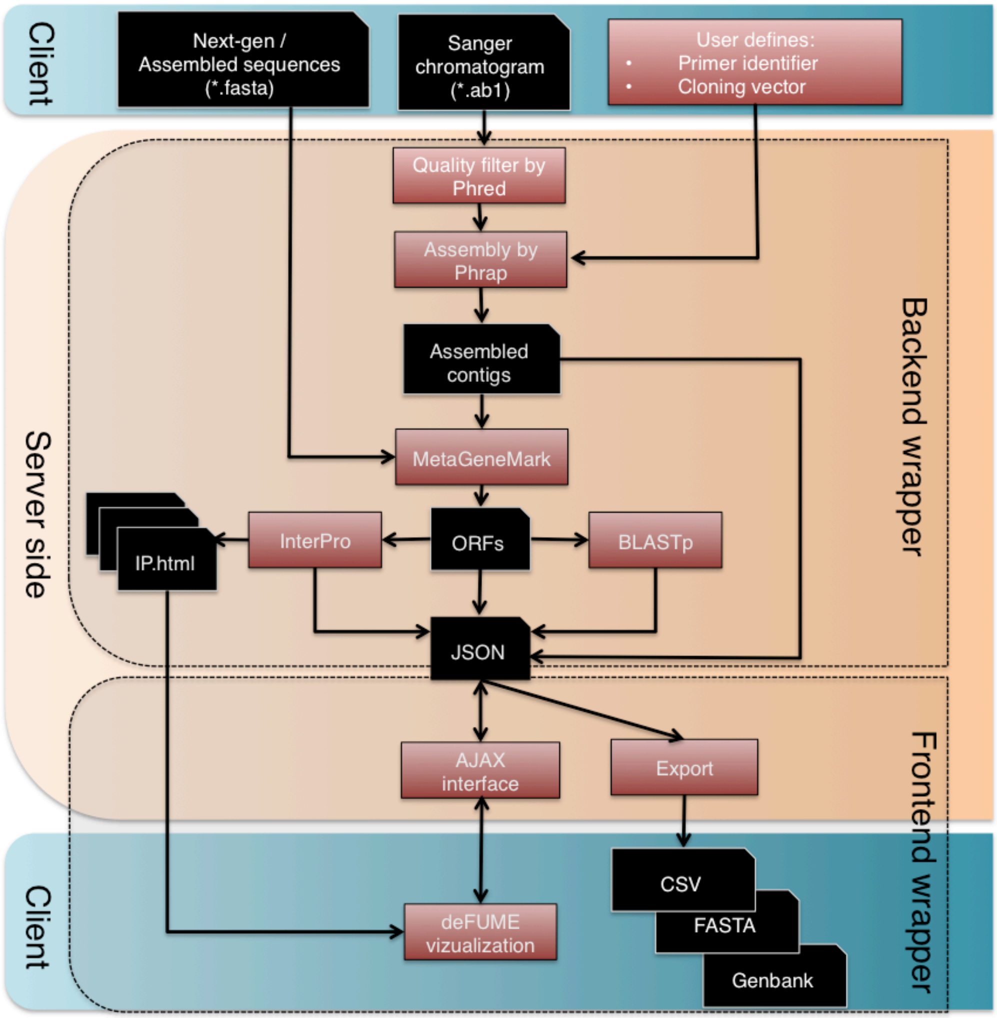

Figure S1. Flowchart of the deFUME webserver, processes are depicted in red and (intermediate) files in black.

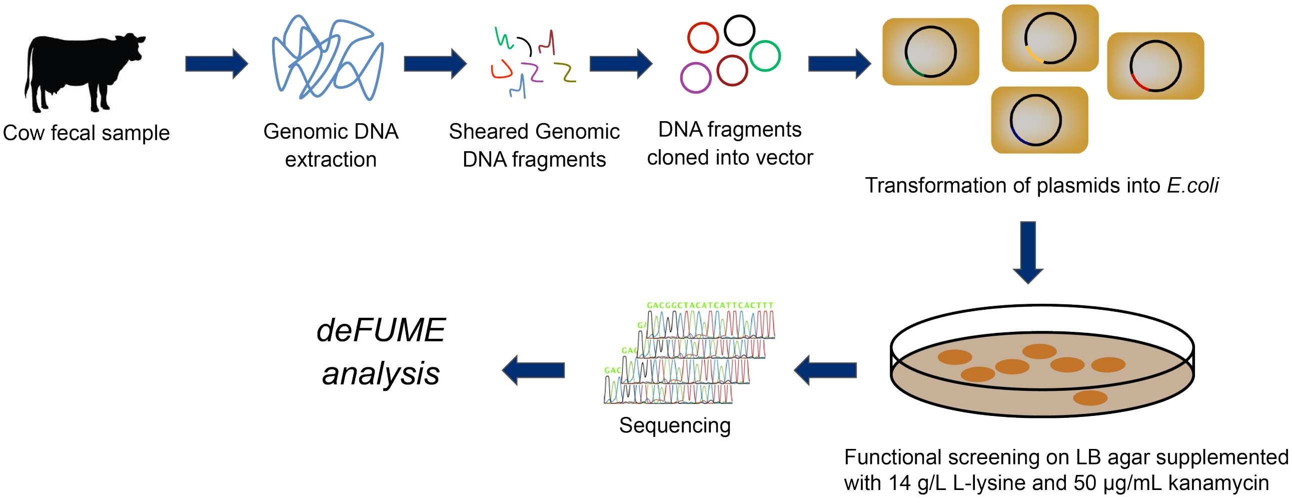

Figure S2: Cow gut microbiota library construction, functional screening for L-lysine tolerant clones and sequence analysis of the metagenomics inserts.

References

1. Sommer MO a, Dantas G, Church GM: **Functional characterization of the antibiotic resistance reservoir in the human microflora.** *Science* 2009, **325**:1128–31.

2. Lutz R, Bujard H: **Independent and tight regulation of transcriptional units in Escherichia coli via the LacR / O , the TetR / O and AraC / I 1 -I 2 regulatory elements**. *Pharmacia* 1997, **25**:1203–1210.
